# Stagnant Neonatal Mortality and Persistent Health Inequality in Middle-Income Countries: A Case Study of the Philippines

**DOI:** 10.1371/journal.pone.0053696

**Published:** 2013-01-07

**Authors:** Aleli D. Kraft, Kim-Huong Nguyen, Eliana Jimenez-Soto, Andrew Hodge

**Affiliations:** 1 School of Economics, University of the Philippines, Quezon City, Philippines; 2 School of Population Health, Public Health Building, Herston Road, Herston, The University of Queensland, Brisbane, Queensland, Australia; University of Massachusetts Medical School, United States of America

## Abstract

**Background:**

The probability of survival through childhood continues to be unequal in middle-income countries. This study uses data from the Philippines to assess trends in the prevalence and distribution of child mortality and to evaluate the country’s socioeconomic-related child health inequality.

**Methodology:**

Using data from four Demographic and Health Surveys we estimated levels and trends of neonatal, infant, and under-five mortality from 1990 to 2007. Mortality estimates at national and subnational levels were produced using both direct and indirect methods. Concentration indices were computed to measure child health inequality by wealth status. Multivariate regression analyses were used to assess the contribution of interventions and socioeconomic factors to wealth-related inequality.

**Findings:**

Despite substantial reductions in national under-five and infant mortality rates in the early 1990s, the rates of declines have slowed in recent years and neonatal mortality rates remain stubbornly high. Substantial variations across urban-rural, regional, and wealth equity-markers are evident, and suggest that the gaps between the best and worst performing sub-populations will either be maintained or widen in the future. Of the variables tested, recent wealth-related inequalities are found to be strongly associated with social factors (e.g. maternal education), regional location, and access to health services, such as facility-based delivery.

**Conclusion:**

The Philippines has achieved substantial progress towards Millennium Development Goal 4, but this success masks substantial inequalities and stagnating neonatal mortality trends. This analysis supports a focus on health interventions of high quality – that is, not just facility-based delivery, but delivery by trained staff at well-functioning facilities and supported by a strong referral system – to re-start the long term decline in neonatal mortality and to reduce persistent within-country inequalities in child health.

## Introduction

Socioeconomic inequalities are gathering momentum as a significant issue beyond the Millennium Development Goal (MDG) deadline in 2015.[1]–[3] There is increasing evidence that substantial inequalities in health across a number of dimensions – including wealth, ethnicity, and geography – continue to exist both between and within countries.[4]–[8] Indeed, it has been suggested that inequalities may have widened in recent times. [9], [10] Persistent inequalities represent a major policy challenge [11] and have prompted a renewed interest in the topic, especially in developing countries.[12]–[15].

The Philippines provides an opportune context to investigate such issues. In spite of achieving an average growth rate in per capita income of approximately three percent over the past decade (2000–10), [16] declines in child mortality rates have faltered. [17] Similarly, the annual rate of decline in maternal mortality has fallen from approximately seven percent over the period 1990–2000 to below one percent for the decade 2000–2011. [18] In pursuit of the MDGs for health, the national government of the Philippines has specified key strategies and reform areas, which explicitly target the poor. [19] Results however have been mixed. For example, improved nutrition has resulted in the stunted-for-age figure declining from nearly one-in-three children under five years of age in 2003 to less than one-in-four in 2005. [20] On the other hand, in recent years there have been limited improvements in maternal and reproductive health and increasing out-of-pocket expenditure. [21].

There is general recognition that priority groups need to be identified with a view of accelerating progress towards the MDGs targets and reducing disparities. Yet, little evidence exists to examine inequalities across several equity-markers and the various country-specific factors associated with such disadvantages.

Whilst anecdotal evidence suggests that differences in health performance exist across urban/rural place of residence, regions, and income status, analysis of the distribution of child health has previously been restricted mostly to wealth. [6], [8] Hence, much is still unknown about the degree of inequalities in the Philippines and the sources of these disparities. To address this gap, and, more generally, to draw policy implications necessary for achieving the MDGs on an equitable basis, we utilise the best available data and examine trends in and distribution of various child health indicators at both the national and subnational levels. In addition we analyse correlations between the inequality of mortality outcomes and the distribution of various risk factors and health care interventions. The present study is the first such analysis in the Philippines.

## Methods

### Ethics Statement

The datasets used in this study were obtained from the MEASURE DHS website, <www.measuredhs.com>. Full review of this study from an institutional review board was not sought as the datasets were anonymous and they are available for public use with no identifiable information on the survey participants.

### Data

The data utilised for this analysis was derived from the Philippines Demographic and Health Surveys (DHS) conducted in 1993, 1998, 2003, and 2008. The DHS collected demographic, socioeconomic, and health data from nationally representative samples of women aged 15–49 years. Two survey instruments were used: a woman’s questionnaire and a household questionnaire. The women (household) samples from the four waves were 15,029 (12,995); 13,983 (12,407); 13,633 (12,586); and 13,594 (12,469), in 1993, 1998, 2003, and 2008, respectively. The surveys employed multi-stage sampling designs (two-stage for 1993 and 1998 and three-stage for 2003 and 2008), which included stratification by region and urban/rural location, and clustering by local settlements called barangays (the primary sampling unit in the Philippines DHS). We examined inconsistencies in survey questions and response options across survey waves and cleaned the data by deleting duplicates and omitting children observations with unfeasible birth and death ages. Further, we validated the data by examining the mortality estimates obtained from different surveys for overlapping periods. If the data were reliable we would expect the levels of mortality computed separately from different surveys to be fairly close to each other for the periods over which the datasets overlap.

The four DHS datasets were utilised to estimate child mortality and wealth-related health inequalities. Regional estimates and the analysis of the contributions of various factors to the measured wealth-related inequality were performed using the most recent DHS wave. All statistical analyses described below were carried out using two statistical packages, *Stata* (version 12) and *R*.

### Mortality Estimates

Child mortality was estimated directly from complete birth histories (CBH), following the methods of Rajaratnam and colleagues [22]. Under-five (U5MR), infant (IMR), and neonatal (NMR) mortality rates were derived from computed survival rates. These rates were estimated using pooled CBH data from all surveys. Due to the relative rarity of deaths, single-year estimates of mortality are not sufficiently precise; hence estimates were made for two-year periods. Loess regression is employed to create a continuous series over time from the biennial estimates, using a smoothing parameter (bandwidth) of 0.5. [23] Confidence intervals on the child mortality estimates are computed using standard simulation methods. [22], [24].

Child mortality is measured at the subnational level using three equity-markers: rural-urban location, regions, and wealth quintiles. Due to data availability, we generate regional estimates using indirect estimation methods [18], [22], [25] on summary birth histories (SBH). Regions are formed via the grouping of fairly homogeneous provinces on the bases of geographical, cultural, and ethnological characteristics. As detailed elsewhere, indirect methods of estimating under-five mortality developed by Rajaratnam and co-authors [22] incorporate cohort-derived and period-derived techniques that use the mother’s age or the time since her first birth. Hence, four methods are available: the time since first birth cohort-derived method (TFBC), the maternal age cohort-derived method (MAC), the time since first birth period-derived method (TFBP), and the maternal age period-derived method (MAP). In order to create a summary measure, we applied Loess regression to the estimates generated from the four methods and capture uncertainty inherent in estimating under-five mortality from SBH following the procedures used by Rajaratnam *et. al.* [22].

Several issues must be addressed to produce regional estimates using these indirect methods. First, these methods run regressions that include country-level random effects. We replace the country-level effect with an island-level effect derived for the Philippines’s three major island groups (i.e. Luzon, Visayas, and Mindanao). Secondly, indirect estimation only delivers estimates of under-five mortality. Thus, one must convert the under-five mortality rates into neonatal and infant mortality rates. To do so, the relationships between the under-five mortality rates and neonatal/infant mortality rates are modelled using a method developed by Murray and colleagues. [26] In short, this process involves transforming under-five, infant, and neonatal mortality rates into logit space; fitting hierarchical models with random intercepts and random slopes at the national and subnational levels; and then using the models to predict neonatal and infant mortality rates from indirect estimates of under-five mortality. One potential drawback of this approach is that by taking into account several sources of uncertainty (i.e. uncertainty from the CBHs, the model itself, and the SBHs estimates) the uncertainty bounds on the resulting infant and neonatal estimates tend to be rather sizable.

Out of sample projections to 2015 were computed following Murray and co-authors. [26] Their method uses the last set of parameter estimates from the Loess regressions to produce the set of forecasts. All estimates take into account the sample weights that come with the data.

### Measuring Wealth-related Health Inequalities

To assess the degree of wealth-related inequality in child mortality we computed the concentration index developed by Wagstaff and co-authors. [27], [28] This index measures the extent to which health status varies between different socioeconomic segments of the population. Given that income and expenditure data are excluded from the DHS, we follow previous studies and compute a wealth index using principal component analysis and questions about household assets and housing characteristics. [29] The general formula for the concentration index (*C*) is: [30]

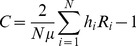
(1)where *h_i_* is the measure of child mortality, *μ* is its mean, *R_i_* is the fractional rank of the *i* individual within the socioeconomic distribution defined by wealth.

For an unbounded variable the index lies between −1 and 1. Its negative values imply that mortality occurs disproportionately among the poorer households, while the opposite is true for its positive values. For bounded variables, such as child mortality, the possible values of the index will be determined by the mean of the distribution and the size of the sample. [31] How to deal with this issue in order to “correct” the concentration index has been hotly debated in recent times.[32]–[34] Two normalisations currently dominate the literature, Wagstaff [31] (i.e. 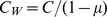) and Erreygers [32], [35] (i.e. 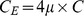).

Erreygers and Van Ourti [36] have recently outlined the various properties of these normalised indices as well as other concentration indices based on the measurement scale and the properties of the underlying health variable. In the case of a binary health variable, they have shown that both the Wagstaff and Erreygers indices possess two desirable properties: the range of the indices vary between −1 and 1, and the ranking of distributions generated by a health variable where good health is denote by a score of 1 is the reverse of the ranking generated by a health variable where good health is denote by a score of 0 (the “mirror property”). The standard concentration index does not satisfy these properties. On the other hand, Erreygers and Van Ourti have also shown that it is the only index that measures relative inequalities, while the Erreygers normalised index captures absolute inequalities and the Wagstaff normalised index measures neither. Given the various properties of the indices and the importance of measuring both relative and absolute inequalities,[37]–[39] we present all three indices.

### Measuring the Contribution of Potential Explanatory Factors to Health Inequalities

For any additive regression of child mortality, the concentration index can be expressed as the sum of the contributions of various explanatory variables associated with child mortality and an unexplained residual component. The choice of concentration index does not affect the interpretation of the decomposition. Each variable’s contribution is the product of (a) the association of child mortality and the explanatory factor and (b) the degree of wealth-related inequality in that factor. For these purposes, we elect to present the decomposition results for the Erreygers normalised index. The decomposition of this index can be written as follows:
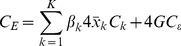
(2)


We link the binary measures of child mortality to a set of covariates using a probit model, with standard errors clustered at the ward (barangays) level. Following van Doorslaer and co-authors [40], we follow a partial effects approach, where the linear approximation uses the partial effects instead of the coefficient parameters. The use of a linear approximation means that the computed contributions are not unique. However, if one computes the average partial effect (APE) for each covariate – that is, one computes the partial effect for each observation in the dataset and then averages these results – and uses these APEs in Equation (2), then the contributions will not, at least, vary across the choice of reference groups.

### Explanatory Factors

The choice of covariates is guided by the conceptual framework of Mosley and Chen [41], as well as previous empirical studies, which have focused on the proximate and socioeconomic factors associated with child mortality.[42]–[44] We investigated the impact of maternal and risk factors, a range of socioeconomic and environmental variables, and interventions that can be delivered through the health sector. The choice of intervention was motivated by numerous studies that demonstrate such interventions to be efficacious against major causes of child mortality at the global level.[45]–[47] The covariates (all categorical) included were: the child’s sex; the birth order of the child (first born and greater than 4); whether the child is a twin/triplet; mother’s pregnancy history (had a terminated pregnancy or a complicated birth), marital status (currently married vs. not); educational level (none vs. primary vs. secondary vs. tertiary); employment status (currently employed vs. not); type of toilet; and health interventions (facility-based birth delivery; if the mother received at least two tetanus toxoid injections; and if the mother uses modern contraception).

We sought to include interventions across the continuum of care from pre-pregnancy to the postpartum period and from the neonatal period to five years of age. However, data on a number of interventions are not available for dead children. For example, exclusive breastfeeding and all measures of vaccination/immunisation variables were not included in the analysis since survey questions corresponding to these measures related only to living children. Moreover, the analysis cannot be undertaken for critical interventions, such as basic-emergency obstetric care, or for risk factors, such as malnutrition, since the relevant data are not available. We also included regional fixed effects to control for unobserved geography-specific effects.

## Results

### Trends and Disparities in Child Mortality

**Figure 1:**
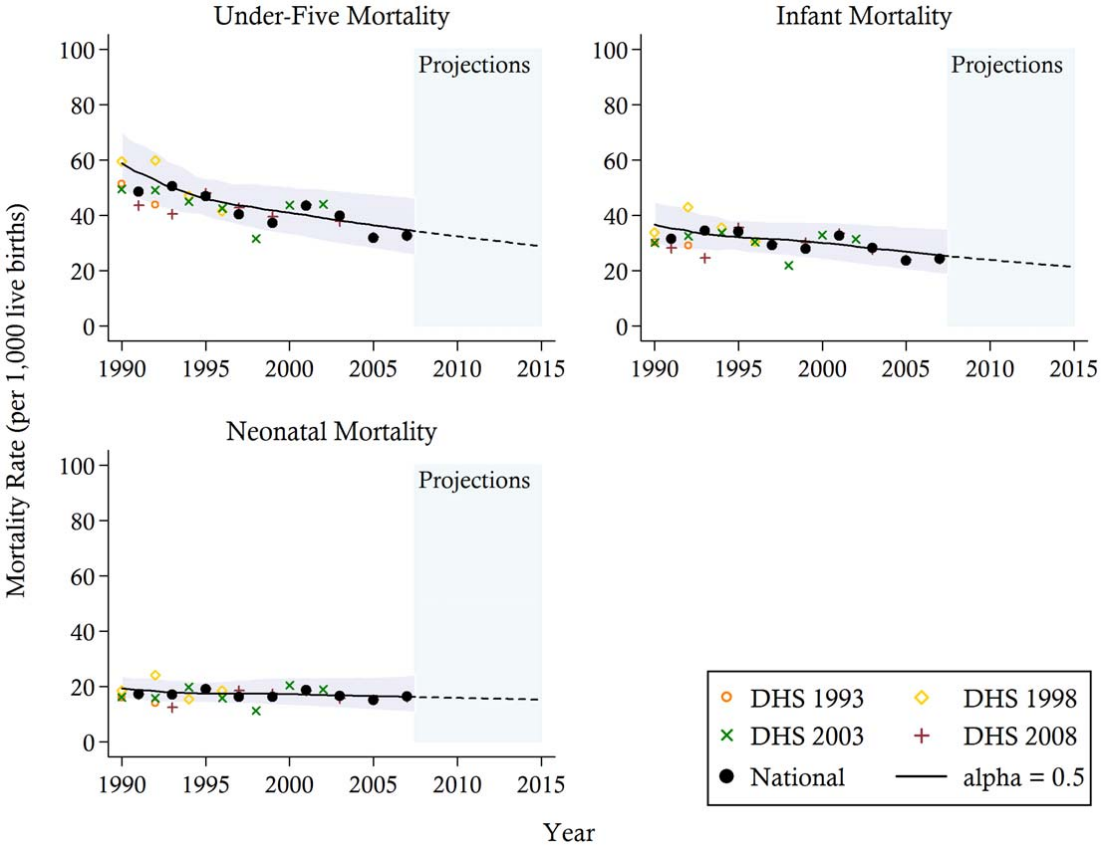
Biennial estimates of under-five, infant, and neonatal mortality rates (per 1,000 live births), with trends between 1990 and 2007 and projections towards 2015 in the Philippines. *Notes*: Data are from Demographic Health Surveys 1993, 1998, 2003, and 2008. Biennial estimates by source and using the pooled data are represented. The solid line represents the mortality estimates calculated using direct methods on the pooled data, while the shaded area signifies the corresponding 95% confidence intervals. DHS, Demographic Health Survey.

Consistent with many large developing countries, our results illustrate that the Philippines has experienced a decline in under-five and infant mortality at the national level. Figure 1 presents the trends and projections to 2015 for rates of neonatal, infant, and under-five mortality. Estimates indicate that the U5MR has fallen from approximately 59 per 1,000 live births (95% CI: 49–70) in 1990 to approximately 34 (95% CI: 26–46) in 2007, while the IMR shows a similar decline from approximately 37 (95% CI: 29–45) in 1990 to 25 (95% CI: 18–35) deaths per 1,000 live births in 2015. The decline in U5MR was sharp in the early 1990s, averaging 4.5 percent per annum, but has weakened since 1996, dropping to half the previous rate of decline to only 2.1 percent. A significant caveat to these declines is the stagnant trend in the neonatal mortality rate, which has remained relatively constant at a level of approximately 18 deaths per 1,000 live births over the entire sample period. These results suggest that neonatal deaths will comprise more than a half of all under-five deaths by 2015 (up from 30 per cent in 1990), and almost three-quarters of infant deaths (up from 48 per cent in 1990).

**Figure 2:**
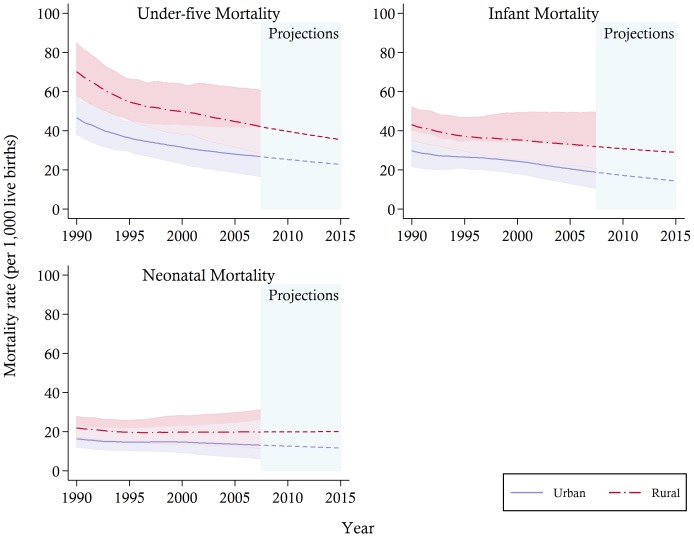
Under-five, infant, and neonatal mortality rates (per 1,000 live births) by rural and urban location: actual 1990–2007; projected to 2015. *Notes*: Data are from Demographic Health Surveys 1993, 1998, 2003, and 2008. The solid and semi-broken lines represent the mortality estimates calculated using direct methods, while the shaded area signifies the corresponding 95% confidence intervals. DHS, Demographic Health Survey.

The pattern of decline in under-five and infant mortality is observed in both urban and rural areas (see Figure 2). It is clear, however, that children residing in urban areas are better off than their rural counterparts. Indeed, the urban under-five mortality in 2007 was already very close to the MDG target. While there is some evidence to suggest that the urban-rural divide is closing slowly in terms of under-five mortality, the gaps in terms of infant and neonatal mortality rates appear to have widened, with both rates dropping faster in the urban areas.

**Figure 3:**
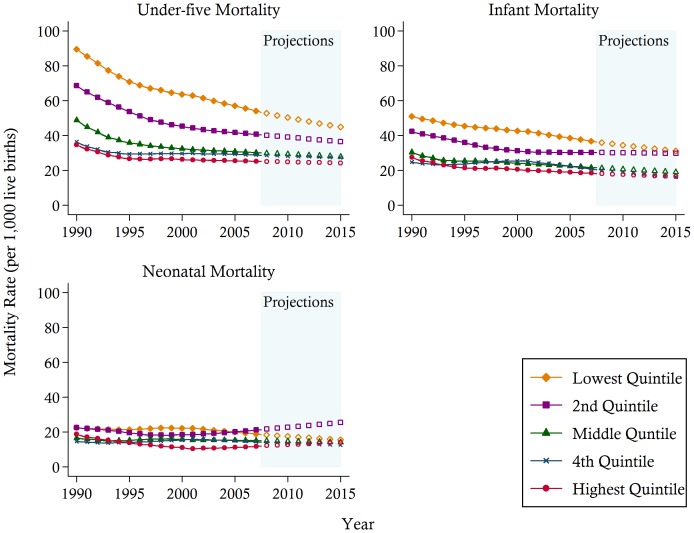
Under-five, infant, and neonatal mortality rates (per 1,000 live births) between 1990 and 2007 and projections towards 2015 in the Philippines by wealth groups. *Notes*: Data are from Demographic Health Surveys 1993, 1998, 2003, and 2008. The solid lines represent the mortality estimates calculated using direct methods. DHS, Demographic Health Survey.

**Figure 4:**
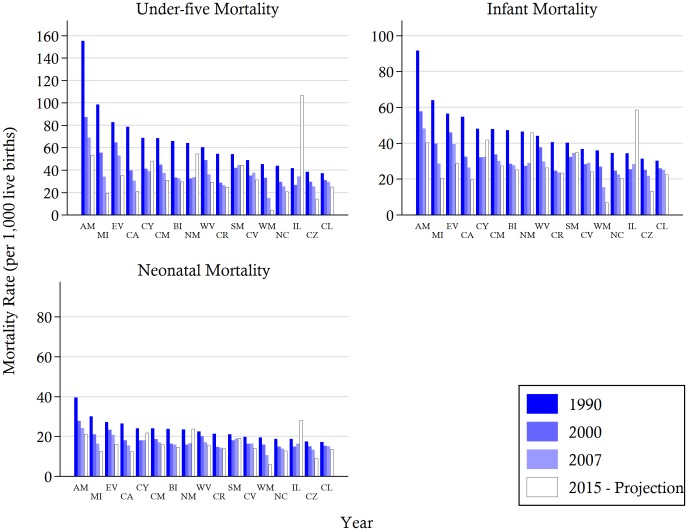
Under-five, infant, and neonatal mortality rates (per 1,000 live births) for selected years and 2015 projections in the Philippines by regions. *Notes*: Data are from Demographic Health Surveys 2008. AM, Autonomous Region in Muslim Mindanao; CR, Cordillera Administrative Region; IL, Ilocoos Region; CY, Cagayan Valley; CL, Central Luzon; CZ, CALABARZON (Cavite, Laguna, Batangas, Rizal, and Quezon); BI, Bicol Region; MI, MIMAROPA (Occidental Mindoro, Oriental Mindoro, Marinduque, Romblon and Palawan); NC, National Capital Region; WV, Western Visayas; CV, Central Visayas; EV, Eastern Visayas; WM, Zamboanga Peninsula (Western Mindanao); NM, Northern Mindanao; SM, Davao Peninsula (Southern Mindanao); CM, SOCCSKSARGEN (South Cotabato, Cotabato, Sultan Kudarat, Sarangani and General Santos City); CA, Caraga Region. The projected increases in mortality rates in the Ilocoos region are driven by estimated upward trends since 2004. Accordingly, the projected trends are driven by a small number of recent estimates, and thus, should be treated with some caution.

The mostly positive national trends mask the substantial unequal progress across wealth groups and regions, as summarised in Figures 3 and 4. These figures show that mortality is more prevalent at the lower end of the socioeconomic spectrum. While there were substantial drops in under-five and infant mortality in the 1990s, especially for the lower quintile groups, the gap between the lowest two wealth quintiles and the upper three wealth quintiles have not narrowed. Under current trends, it is likely that the upper three quintiles will achieve convergence but the lowest two quintiles will remain far behind. A worrying trend is the levelling off in the decline of child mortality of those households belonging to the second lowest quintile, and the apparent upward trend in their neonatal mortality rate since the mid-1990s.

**Figure 5:**
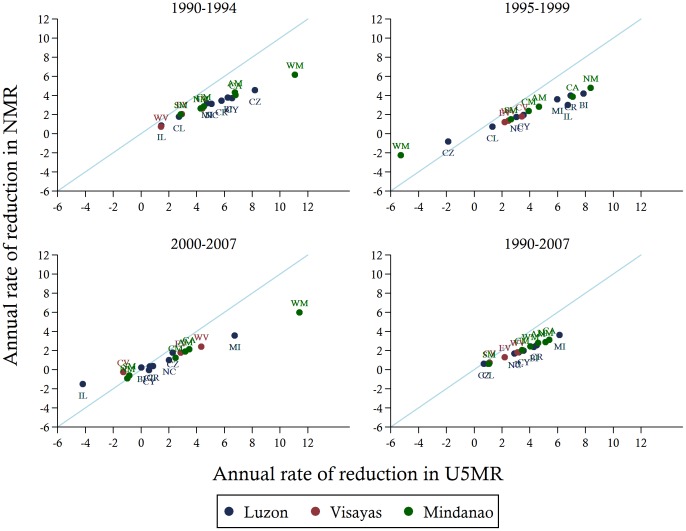
Annual rates of reduction in under-five vs. neonatal mortality rates over the periods 1990–1994, 1995–1999, 2000–2007, and 1990–2007 in the Philippines by regions. *Notes*: Data are from Demographic Health Surveys 2008. AM, Autonomous Region in Muslim Mindanao; CR, Cordillera Administrative Region; IL, Ilocoos Region; CY, Cagayan Valley; CL, Central Luzon; CZ, CALABARZON (Cavite, Laguna, Batangas, Rizal, and Quezon); BI, Bicol Region; MI, MIMAROPA (Occidental Mindoro, Oriental Mindoro, Marinduque, Romblon and Palawan); NC, National Capital Region; WV, Western Visayas; CV, Central Visayas; EV, Eastern Visayas; WM, Zamboanga Peninsula (Western Mindanao); NM, Northern Mindanao; SM, Davao Peninsula (Southern Mindanao); CM, SOCCSKSARGEN (South Cotabato, Cotabato, Sultan Kudarat, Sarangani and General Santos City); CA, Caraga Region; NMR, neonatal mortality rate; U5MR, under-five mortality rate.

The disaggregation of under-five, infant, and neonatal mortality rates by regions illustrates the gaps in child mortality across the different geographical locations and suggests that many are likely to worsen by 2015. As shown in Figure 5, for most regions, the rate of decline in NMR is slower than the rate of decline in U5MR. Moreover, the rates of reduction vary within the three main geographical divisions of the Philippines. The national downward trend in child mortality is not uniform throughout the country, with some regions reducing child mortality faster than others and some experiencing an upward trend. For example, four regions (Ilocos, Central Visayas, Northern Mindanao, and Davao Penninsula) have experienced negative annual rates of reduction in both under-five and neonatal mortality over the recent 2000–07 period. In other regions, however, the downward trends in mortality have been sustained, with some wealthier regions, such as the National Capital Region (NCR) and Cavite, Laguna, Batangas, Rizal, and Quezon (CALABARZON) region, achieving mortality rates matching the MDG targets. Nonetheless, a number of regions continue to lag behind and widening disparities across geographically units are observed.

### Levels of Wealth-relate Health Inequality and Contributions

Within-country inequalities in mortality rates are also found amongst socioeconomic strata at the national level and for urban\rural sub-populations. The results are summarised in Table 1, which presents the corresponding concentration indexes. At both the national and subnational levels, the burden of neonatal, infant (not shown), and under-five mortality is disproportionately borne by the poor. This has remained true over the period 1993 to 2008. At the national level, the degree of inequality is more severe for under-five mortality than for infant and neonatal deaths, respectively. Furthermore, we see that the unequal distribution of mortality remained largely unchanged, with some slight improvements for under-five mortality. In combination with the trends discussed above, these results imply that reductions in mortality have been somewhat biased in favour of the rich. A downward trend in inequality over time is observed in rural areas, while inequality in under-five mortality shows some signs of rising slightly in urban locations.

**Table 1:** Concentration indices of wealth-related inequalities in under-five and neonatal mortality, Philippines 1993–2008.

	Under-five Mortality						Neonatal Mortality					
	CI	s.e.	WCI	s.e.	ECI	s.e.	CI	s.e.	WCI	s.e.	ECI	s.e.
**National**												
DHS 1993	−0.2160	(0.029)	−0.2248	(0.030)	−0.0338	(0.005)	−0.0936	(0.043)	−0.0956	(0.044)	−0.0077	(0.004)
DHS 1998	−0.2167	(0.035)	−0.2250	(0.036)	−0.0321	(0.005)	−0.1528	(0.050)	−0.1558	(0.051)	−0.0121	(0.004)
DHS 2003	−0.1618	(0.037)	−0.1677	(0.038)	−0.0225	(0.005)	−0.0906	(0.051)	−0.0924	(0.052)	−0.0070	(0.004)
DHS 2008	−0.1943	(0.044)	−0.1998	(0.045)	−0.0214	(0.005)	−0.1217	(0.056)	−0.1239	(0.057)	−0.0088	(0.004)
**Rural**												
DHS 1993	−0.2035	(0.033)	−0.2133	(0.035)	−0.0375	(0.007)	−0.1410	(0.050)	−0.1442	(0.051)	−0.0127	(0.005)
DHS 1998	−0.1552	(0.042)	−0.1621	(0.044)	−0.0265	(0.007)	−0.1045	(0.062)	−0.1068	(0.063)	−0.0092	(0.005)
DHS 2003	−0.1155	(0.046)	−0.1204	(0.048)	−0.0189	(0.008)	0.0013	(0.071)	0.0014	(0.072)	0.0001	(0.006)
DHS 2008	−0.0924	(0.056)	−0.0955	(0.057)	−0.0121	(0.007)	−0.0559	(0.071)	−0.0571	(0.072)	−0.0048	(0.006)
**Urban**												
DHS 1993	−0.1542	(0.049)	−0.1592	(0.050)	−0.0195	(0.006)	0.0083	(0.062)	0.0084	(0.063)	0.0006	(0.005)
DHS 1998	−0.2489	(0.062)	−0.2567	(0.064)	−0.0303	(0.008)	−0.1907	(0.083)	−0.1941	(0.085)	−0.0131	(0.006)
DHS 2003	−0.1646	(0.056)	−0.1695	(0.057)	−0.0189	(0.007)	−0.1854	(0.075)	−0.1886	(0.076)	−0.0127	(0.005)
DHS 2008	−0.2432	(0.078)	−0.2487	(0.080)	−0.0214	(0.007)	−0.0943	(0.096)	−0.0957	(0.098)	−0.0055	(0.006)

*Notes*: Total number of observations for the 1993, 1998, 2003, and 2008 survey waves were 9,195; 8,083; 7,145; and 6,572, respectively. CI, concentration index; WCI, Wagstaff-normalised concentration index; ECI, Erreygers-normalised concentration index; s.e., standard error.

The associations and contributions of various explanatory factors to wealth-related inequality are reported for under-five and neonatal mortality. To conserve space, we do not report the results for infant mortality, which are similar to the under-five mortality results. The associations between under-five mortality and its potential determinants are shown in Table 2. As expected, the risk factors are all associated with a greater probability of under-five mortality, although only a history of a complicated birth and multiple births are found to be statistically significant. Similarly, amongst the indicators of socioeconomic status, only a more highly educated mother is found to be associated with lower risk of under-five mortality. Lastly, in terms of the health interventions, facility-based delivery and the mother having had at least two tetanus injections during pregnancy show a negative association with under-five mortality.

**Table 2:** Decomposition analysis of inequality in under-five mortality, Philippines 2008.

Variable	Coeff.		S.E.	Mean	P.E.	S.E.	C.	S.E.	Cont.	% Cont.
Maternal/Risk factors										
First born	0.0371		(0.125)	0.2796	0.0016	(0.006)	0.1620	(0.014)	0.0003	−2.04
Birth order >4	0.1727		(0.108)	0.2047	0.0080	(0.005)	−0.3139	(0.015)	−0.0021	14.33
Multiple births	0.7469	**	(0.297)	0.0074	0.0639	(0.042)	0.0200	(0.109)	0.00004	−0.26
Term. preg.	0.0720		(0.106)	0.2015	0.0032	(0.005)	−0.0994	(0.017)	−0.0003	1.78
Comp. birth	0.2500	**	(0.101)	0.2671	0.0119	(0.005)	0.0479	(0.015)	0.0006	−4.24
Female	−0.0563		(0.096)	0.4718	−0.0024	(0.004)	0.0051	(0.009)	−0.00002	0.16
Socioecon./Env. char.										
Married	−0.1515		(0.102)	0.7267	−0.0069	(0.005)	−0.0119	(0.005)	0.0002	−1.66
E: None	[Base]									
E: Primary	−0.5494	***	(0.185)	0.2324	−0.0341	(0.017)	−0.4285	(0.014)	0.0136	
E: Secondary	−0.3580	*	(0.190)	0.4786	−0.0259	(0.018)	−0.0031	(0.009)	0.0002	
E:Teritary	−0.6552	***	(0.234)	0.2739	−0.0375	(0.018)	0.4106	(0.011)	−0.0169	21.68
Mother Employed	0.0907		(0.099)	0.4141	0.0040	(0.004)	0.0710	(0.011)	0.0005	−3.23
Toilet	−0.0828		(0.299)	0.0405	−0.0033	(0.011)	0.0591	(0.042)	−0.00003	0.22
Interventions										
FBD	−0.2976	***	(0.114)	0.4655	−0.0120	(0.005)	0.3002	(0.008)	−0.0067	46.69
TT2 plus	−0.2182	**	(0.104)	0.4808	−0.0091	(0.004)	0.0663	(0.009)	−0.0012	8.05
Modern cont.	−0.0151		(0.102)	0.3450	−0.0006	(0.004)	0.0809	(0.012)	−0.0001	0.50
Regions										
R: ARMM	[Base]									
R: Ilocos Region	−0.2522		(0.276)	0.0475	−0.0112	(0.012)	0.1941	(0.032)	−0.0004	
R: Cagayan Valley	0.0711		(0.273)	0.0313	0.0043	(0.017)	−0.0932	(0.037)	−0.00005	
R: Central Luzon	0.0568		(0.207)	0.1016	0.0034	(0.012)	0.1936	(0.023)	0.0003	
R: Bicol Region	−0.4038		(0.310)	0.0619	−0.0156	(0.011)	−0.1957	(0.032)	0.0008	
R: Western Visayas	−0.0155		(0.202)	0.0713	−0.0009	(0.011)	−0.1868	(0.034)	0.00005	
R: Central Visayas	−0.0173		(0.221)	0.0718	−0.0010	(0.012)	−0.0367	(0.034)	0.00001	
R: Eastern Visayas	0.1891		(0.222)	0.0429	0.0127	(0.016)	−0.3056	(0.034)	−0.0007	
R: Z. Pen.	−0.4220		(0.293)	0.0413	−0.0161	(0.010)	−0.2508	(0.037)	0.0007	
R: N. Mindanao	−0.2395		(0.243)	0.0435	−0.0108	(0.011)	−0.2014	(0.039)	0.0004	
R: Davao Peninsula	−0.1356		(0.254)	0.0487	−0.0067	(0.012)	−0.1584	(0.034)	0.0002	
R: C. Mindanao	−0.1591		(0.252)	0.0388	−0.0077	(0.012)	−0.2586	(0.034)	0.0003	
R: Caraga Region	−0.3440		(0.258)	0.0272	−0.0141	(0.010)	−0.2095	(0.032)	0.0003	
R: NCR	−0.1905		(0.238)	0.1477	−0.0090	(0.011)	0.3669	(0.018)	−0.0019	
R: CAR	−0.0092		(0.262)	0.0160	−0.0005	(0.015)	−0.0174	(0.039)	0.000001	
R: CALABARZON	−0.6527	**	(0.290)	0.1300	−0.0203	(0.009)	0.2975	(0.021)	−0.0031	
R: MIMAROPA	0.0101		(0.262)	0.0331	0.0006	(0.015)	−0.2981	(0.035)	−0.00002	22.70
Constant	−1.3520	***	(0.241)							
Residual									0.0007	−4.69
Obs. (PSU)	4659 (773)									
Log likelihood	−389.107									
Pseudo R^2^	0.083									

*Notes:* Coeff., coefficient; S.E., standard error; P.E., partial effect; C., concentration index; Cont., contribution; % Cont., percentage contribution; Term. Preg., terminated pregnancy; Comp. birth, complicated birth; Socioecon./Env. char., Socioeconomic and environmental characteristics; FBD, facility-based delivery; TT2 plus, at least two tetanus toxoid; Modern cont., modern contraception; ARMM Autonomous Region in Muslim Mindanao; Z. Pen Zamboanga Peninsula; N. Mindanao, Northern Mindanao; C. Mindanao, Central Mindanao; NCR, National Capital Region; CAR, Cordillera Administrative Region; CALABARZON, Cavite, Laguna, Batangas, Rizal, and Quezon; MIMAROPA, Occidental Mindoro, Oriental Mindoro, Marinduque, Romblon and Palawan. PSU, primary sampling unit. (***), (**), (*) indicate p<0.01, p<0.05, p<0.1 respectively.

The extent to which each of the covariates is unequally distributed by socioeconomic status is reflected by their corresponding concentration indices. For example, mothers’ tertiary level of education, coverage of facility-based delivery, and tetanus injections are more concentrated amongst the rich. In contrast, indicators of high birth order and terminated pregnancies are more concentrated amongst the poor.

The contribution of each of the covariates to wealth-related inequality in under-five mortality is also reported in Table 2. For ease of interpretation, the percentage contribution shows the grouped contribution for the multi-category variables. A negative contribution implies that the variable is lowering wealth inequality and vice versa. Our findings suggest that of the variables tested the largest contributions to inequality in under-five mortality were attributable to facility-based delivery (46.7%), the regional fixed effects (22.7%), and the mother’s education (21.7%). Furthermore, higher birth order (14.3%) and tetanus injections (8%) show a noteworthy contribution to the measured inequality. It is reassuring to note that the contribution of the generalised residual only amounted to approximately four percent, indicating that the model functioned well.

As expected, since a large proportion of under-five deaths occur amongst neonates, similar variables were found to contribute to wealth-related inequality in neonatal mortality (see Table 3). Again the largest contributors are facility-based delivery (74.1%) and the mother’s education (23.2%), with higher birth order (17.5%) and tetanus injections (17.3%) remaining substantial. For both under-five and neonatal mortality, the large contribution of facility-based delivery is a combination of a substantial association with reducing child mortality and a considerable wealth-related gradient.

**Table 3:** Decomposition analysis of inequality in neonatal mortality, Philippines 2008.

Variable	Coeff.		S.E.	Mean	P.E.	S.E.	C.	S.E.	Cont.	% Cont.
Maternal/Risk factors										
First born	0.1668		(0.153)	0.2796	0.0052	(0.005)	0.1620	(0.014)	0.0009	−16.49
Birth order >4	0.1280		(0.135)	0.2047	0.0039	(0.004)	−0.3139	(0.015)	−0.0010	17.54
Multiple births	1.0100	***	(0.300)	0.0074	0.0793	(0.045)	0.0200	(0.109)	0.00005	−0.82
Term. preg.	0.1751		(0.126)	0.2015	0.0055	(0.004)	−0.0994	(0.017)	−0.0004	7.71
Comp. birth	0.2405	**	(0.123)	0.2671	0.0077	(0.004)	0.0479	(0.015)	0.0004	−6.83
Female	−0.1591		(0.119)	0.4718	−0.0045	(0.003)	0.0051	(0.009)	−0.00004	0.75
Socioecon./Env. char.										
Married	0.0262		(0.125)	0.7267	0.0007	(0.003)	−0.0119	(0.005)	−0.00003	0.44
E: None	[Base]									
E: Primary	−0.8292	***	(0.232)	0.2324	−0.0421	(0.020)	−0.4285	(0.014)	0.0168	
E: Secondary	−0.5779	**	(0.225)	0.4786	−0.0355	(0.021)	−0.0031	(0.009)	0.0002	
E:Teritary	−0.7638	***	(0.268)	0.2739	−0.0408	(0.021)	0.4106	(0.011)	−0.0183	23.21
Mother Employed	0.1835		(0.119)	0.4141	0.0054	(0.004)	0.0710	(0.011)	0.0006	−11.03
Toilet	−0.1277		(0.381)	0.0405	−0.0032	(0.008)	0.0591	(0.042)	−0.00003	0.54
Interventions										
FBD	−0.2743	**	(0.136)	0.4655	−0.0076	(0.004)	0.3002	(0.008)	−0.0043	74.09
TT2 plus	−0.2819	**	(0.124)	0.4808	−0.0078	(0.003)	0.0663	(0.009)	−0.0010	17.27
Modern cont.	0.0694		(0.119)	0.3450	0.0020	(0.004)	0.0809	(0.012)	0.0002	−3.95
Regions										
R: ARMM/Z. Pen.	[Base]									
R: Ilocos Region	0.1273		(0.314)	0.0475	0.0026	(0.007)	0.1941	(0.032)	0.0001	
R: Cagayan Valley	0.3845		(0.288)	0.0313	0.0108	(0.010)	−0.0932	(0.037)	−0.0001	
R: Central Luzon	0.5139	**	(0.240)	0.1016	0.0169	(0.010)	0.1936	(0.023)	0.0013	
R: Bicol Region	0.0236		(0.294)	0.0619	0.0004	(0.005)	−0.1957	(0.032)	−0.00002	
R: Western Visayas	0.3032		(0.221)	0.0713	0.0077	(0.006)	−0.1868	(0.034)	−0.0004	
R: Central Visayas	0.2964		(0.233)	0.0718	0.0075	(0.007)	−0.0367	(0.034)	−0.0001	
R: Eastern Visayas	0.4019	*	(0.239)	0.0429	0.0115	(0.008)	−0.3056	(0.034)	−0.0006	
R: N. Mindanao	0.0082		(0.312)	0.0435	0.0001	(0.006)	−0.2014	(0.039)	−0.00001	
R: Davao Peninsula	0.1608		(0.310)	0.0487	0.0035	(0.007)	−0.1584	(0.034)	−0.0001	
R: C. Mindanao	−0.1939		(0.370)	0.0388	−0.0028	(0.005)	−0.2586	(0.034)	0.0001	
R: Caraga Region	0.0571		(0.297)	0.0272	0.0011	(0.006)	−0.2095	(0.032)	−0.00002	
R: NCR	0.3543		(0.241)	0.1477	0.0096	(0.007)	0.3669	(0.018)	0.0021	
R: CAR	0.4982	*	(0.271)	0.0160	0.0160	(0.012)	−0.0174	(0.039)	−0.00002	
R: CALABARZON	−0.3093		(0.370)	0.1300	−0.0039	(0.004)	0.2975	(0.021)	−0.0006	
R: MIMAROPA	0.2735		(0.273)	0.0331	0.0067	(0.008)	−0.2981	(0.035)	−0.0003	−23.54
Constant	−1.8773	***	(0.262)							
Residual									−0.0012	21.11
Obs. (PSU)	4659 (773)									
Log likelihood	−264.407									
Pseudo R^2^	0.094									

*Notes:* Coeff., coefficient; S.E., standard error; P.E., partial effect; C., concentration index; Cont., contribution; % Cont., percentage contribution; Term. Preg., terminated pregnancy; Comp. birth, complicated birth; Socioecon./Env. char., Socioeconomic and environmental characteristics; FBD, facility-based delivery; TT2 plus, at least two tetanus toxoid; Modern cont., modern contraception; ARMM, Autonomous Region in Muslim Mindanao; Z. Pen, Zamboanga Peninsula; N. Mindanao, Northern Mindanao; C. Mindanao, Central Mindanao; NCR, National Capital Region; CAR, Cordillera Administrative Region; CALABARZON, Cavite, Laguna, Batangas, Rizal, and Quezon; MIMAROPA,Occidental Mindoro, Oriental Mindoro, Marinduque, Romblon and Palawan. PSU, primary sampling unit. (***), (**), (*) indicate p<0.01, p<0.05, p<0.1 respectively.

## Discussion

In pursuit of accelerated progress towards the MDGs it is plausible that some disadvantaged groups will be left behind. [9] This study demonstrates that important within-country inequalities in child health outcomes remain in the Philippines. Child mortality rates vary substantially across a number of dimensions, including rural/urban location, groupings of provinces, and wealth status. The gaps between the best and worst performing sub-populations persist, and in some cases, are found to be increasing over time and are projected to widen.

Notwithstanding the acceleration in under-five mortality reductions observed across Southeast Asia [48], our results show that this is not the case for the Philippines. The national annual rate of decline has slowed down in recent years; from approximately 3.7% over the period 1990–99 to 2.3% over 2000–07. Additionally, less progress has been made for neonatal mortality, which has remained relatively constant since the 1990s. A pattern of slower decline in neonatal mortality relative to older age groups and the corresponding increase in the proportion of under-five deaths occurring in the neonatal period has been observed globally. [18], [48] Substantial progress in neonatal mortality would thus require well-targeted interventions addressing the major causes of neonatal mortality in the worst-performing groups.

Our results also suggest that children with lower socioeconomic status are less likely to survive childhood. Convergence in mortality rates is expected for the upper three quintiles but the lowest two quintiles would remain far behind. Additionally, the seemingly upward trend in mortality, especially for neonatal mortality, of those belonging to the second lowest quintile, indicates that, while the government may be achieving some success in reaching the poorest of the poor, the “near-poor” might be missing out. These findings suggest that efforts to target the poor should also consider including the “near-poor”, whose living conditions, risk factors, and barriers to care may not be substantially different to those in the bottom quintile.

As has been seen in other countries where health services have been devolved to local authorities, the observed disparities at the regional level point to variations in the performance of local governments. [7] Lack of health professionals and their unequal distribution, low investments in health sector infrastructure, and geographical inaccessibility to health facilities in remote locations seem to be some of the underlying factors. [21] However, it should also be noted that extreme poverty and persistent armed conflict may also be important factors. Those regions that have reduced mortality rates most successfully – for example, NCR and CALABARZON – appear also to be the wealthier regions.

The importance of wealth-related inequality is confirmed at the individual level, where estimated concentration indices highlight that mortality is borne more by poor households. The analysis of the contribution of available potential determinants of child mortality to wealth-related inequality draws attention to a number of important factors. Based on the percentage contributions to overall socioeconomic inequality in child mortality, it is clear that most of the inequality is associated with the delivery of health services, as proxied by facility-based delivery, and social determinants in the form of the mother’s education. Maternal and risk factors, including high birth order, if a child is a twin or one in a larger multiple birth, and children of mothers with histories of complicated births were also found to be associated with an increase in under-five and neonatal mortality risk. The latter two factors’ contributions to inequality in child mortality, however, are trivial due to their relatively equal concentration across wealth quintiles.

The link between facility-based delivery and socioeconomic inequality is an important result. Facility-based delivery has not only been identified as an effective intervention for the prevention of neonatal mortality, but it is also a complex intervention that requires a functioning health system capable of providing 24-hour functioning facilities with trained staff, as well as being a component of emergency neonatal care and comprehensive emergency obstetric care. [49] Consequently, it captures dimensions of the quality of the health system and reinforces the notion of the importance of addressing inequalities in the delivery of health services in achieving the MDGs. [50] Similarly, the noteworthy association between the mother having had at least two tetanus injections and child mortality again highlights the significance of effective interventions. This variable may be capturing access to antenatal care since tetanus toxoid injections are most likely given during these visits, and may also act as a partial proxy for quality antenatal care, given that it requires available supplies and logistics to enable vaccination.

Region of residence also figures prominently as a contributor to socioeconomic inequality in child mortality. A majority of the contribution comes from the influence of the wealthy regions (i.e. NCR and CALABARZON), where there is a high concentration of the economically better off section of the society. While we do not directly test the impact of persistent conflict in the south, the regional estimates in Figure 4 suggest its potential influence on child mortality. The Autonomous Region in Muslim Mindanao (ARMM) is found to have the highest levels of under-five, infant, and neonatal mortality amongst all provinces. Similarly, the poor southern regions of South Cotabato, Cotabato, Sultan Kudarat, Sarangani and General Santos City (SOCSKSARGEN) and Carga were found to be in the bottom 6 regions based on the 1990 estimates. However, while the average gap may be wider with the inclusion of the southern regions, removing them would not erase the regional gaps in mortality rates. Yet, we should note that given the available data, we are not able to disentangle the effects of “region of residence” and other factors such as economic development or civil conflict.

The considerable contribution of the education of the mother to the socioeconomic inequalities in child survival is consistent with the few past studies on health inequalities in developing countries. [12], [51], [52] It has been suggested that maternal education can influence the child survival via life style choices that improve the ability of individuals to maintain and improve their health and that of their family (e.g. awareness of the importance of early breastfeeding) as well as motivating the increased use of health care services through knowledge and affecting attitudes and practice. [52], [53] Our results are supportive of such notions and imply continual need for educational campaigns to be directed equally throughout the country.

Finally, several caveats relate to the findings of this study. First, the available data imposed several limitations on the analysis of mortality and intervention coverage. Insufficient data were available to estimate mortality rates at lower levels of public administration. Based on important disparities found across regions, a strong rationale exists to undertake the analysis at lower subnational units when the necessary data becomes available. Furthermore, data on some highly relevant interventions are not available in the DHS datasets. For example, questions specifically addressing either pneumonia or malaria, two major causes of death in children under-five, were not available. Moreover, the datasets do not contain questions that directly capture dimensions of the quality of care provided by childhood health services. This necessitated the use of questions on specific service content, which at best can only be used as indirect proxies for service quality. Secondly, our forecasts are based on recent time trends, and consequently we do not control for the possible impacts of intensified efforts to reduce child mortality in certain areas or by targeting specific sub-populations. Thirdly, the use of cross-sectional data prevents us from firmly establishing robust causal relationships. The issue of causality might be better investigated from the use of longitudinal or experimental data. Finally, the analysis of the contribution of explanatory factors to the concentration index is limited to linear models. Since a nonlinear model is used, the analysis required the use of a linear approximation. The use of partial effects for this purpose means that the analysis is not unique. Furthermore, the analysis relies on the specification of the underlying model to be appropriate. In our case, we relied on both the results of previous studies and a well-established conceptual framework to ensure the robustness of our results.

In conclusion, in this study we have sought to estimate the levels of, and inequalities and trends in, child mortality. Furthermore, we have examined a range of proximate and socioeconomic potential determinants of such inequalities. While it is evident that the Philippines, like many other middle-income countries, has achieved progress towards MDG 4, neonatal mortality remains stagnant and greater efforts should be placed on its reduction. Concurrently, success at the national level in under-five and infant mortality reduction masks inequality in progress at the subnational level. Persistent inequality in mortality outcomes across geographical location and socioeconomic status is evident, and the burden of mortality is mostly borne by the poor. Wealth-related inequality remains large and appears to be strongly associated with important health services and social determinants. The unequal distribution of health interventions, particularly facility-based delivery and tetanus toxoid, is found to contribute to inequalities in child deaths, suggesting that barriers to utilisation remain. Many development challenges are faced by middle-income countries. As such countries take strides in reducing child mortality in absolute levels and in reducing the comparative disadvantage experienced by its most vulnerable groups, the results of this study demonstrate that disparities and inequalities persist across the sub-populations of one such country. Future improvements will increasingly rely on the more difficult task of strengthening health systems, and continued efforts will be needed to capitalise on, and amplify, past gains.
